# Gene Expression Response of *Salmonella enterica* Serotype Enteritidis Phage Type 8 to Subinhibitory Concentrations of the Plant-Derived Compounds *Trans*-Cinnamaldehyde and Eugenol

**DOI:** 10.3389/fmicb.2017.01828

**Published:** 2017-09-26

**Authors:** Anup Kollanoor Johny, Jonathan G. Frye, Annie Donoghue, Dan J. Donoghue, Steffen Porwollik, Michael McClelland, Kumar Venkitanarayanan

**Affiliations:** ^1^Department of Animal Science, University of Minnesota, Saint Paul, MN, United States; ^2^Bacterial Epidemiology and Antimicrobial Resistance Research Unit, USDA-ARS, Richard B. Russell Research Center, Athens, GA, United States; ^3^Poultry Production and Product Safety Research Unit, USDA, Fayetteville, AR, United States; ^4^Department of Poultry Science, University of Arkansas, Fayetteville, AR, United States; ^5^Department of Microbiology and Molecular Genetics, University of California, Irvine, Irvine, CA, United States; ^6^Department of Animal Science, University of Connecticut, Storrs, CT, United States

**Keywords:** plant-derived, *trans*-cinnamaldehyde, eugenol, *Salmonella* Enteritidis PT8, microarray, antibacterial

## Abstract

**Background:** *Salmonella* Enteritidis phage type 8 (PT8) is a major poultry-associated *Salmonella* strain implicated in foodborne outbreaks in the United States. We previously reported that two plant-derived compounds generally recognized as safe (GRAS), *trans*-cinnamaldehyde (TC), and eugenol (EG), significantly reduced *S*. Enteritidis colonization in broiler and layer chickens. To elucidate potential PT8 genes affected by TC and EG during colonization, a whole-genome microarray analysis of the bacterium treated with TC and EG was conducted.

**Results:** *S*. Enteritidis PT8 was grown in Luria-Bertani broth at 37°C to an OD_600_ of ~0.5. Subinhibitory concentrations (SICs; concentration that does not inhibit bacterial growth) of TC (0.01%; 0.75 mM) or EG (0.04%; 2.46 mM) were then added to the culture. *S*. Enteritidis PT8 RNA was extracted before and 30 min after TC or EG addition. Labeled cDNA from three replicate experiments was subsequently hybridized to a microarray of over 99% of *S*. Enteritidis PT4 genes, and the hybridization signals were quantified. The plant-derived compounds down-regulated (*P* < 0.005) expression of *S*. Enteritidis PT8 genes involved in flagellar motility, regulation of the *Salmonella* Pathogenicity Island 1, and invasion of intestinal epithelial cells. TC and EG also suppressed transcription of genes encoding multiple transport systems and outer membrane proteins. Moreover, several metabolic and biosynthetic pathways in the pathogen were down-regulated during exposure to the plant-derived compounds. Both TC and EG stimulated the transcription of heat shock genes, such as *dnaK, dnaJ, ibpB*, and *ibpA* in *S*. Enteritidis PT8 (*P* < 0.005). The results obtained from microarray were validated using a quantitative real-time PCR.

**Conclusion:** The plant-derived compounds TC and EG exert antimicrobial effects on *S*. Enteritidis PT8 by affecting multiple genes, including those associated with virulence, colonization, cell membrane composition, and transport systems.

## Background

*Salmonella enterica* serovar Enteritidis (*S*. Enteritidis) is one of the most commonly isolated *Salmonella* serotypes from poultry (Centers for Disease Control and Prevention, 2010; Campioni et al., 2013; Gould et al., 2013), and is responsible for about one third of the reported human salmonellosis outbreaks in the United States (Gould et al., 2013). The pathogen poses a significant health concern for human health due to the contamination of poultry meat and eggs, which constitute the most common food products linked to human salmonellosis (Guard-Petter, 2001; Marcus et al., 2007). In chickens, the cecum is the most common site for *Salmonella* residency (Gantois et al., 2009; Kollanoor Johny et al., 2012a,b,c). The cecal colonization by the pathogen results in fecal shedding, invasion of reproductive organs, contamination of egg shells and yolks, and carcass contamination during slaughter (Keller et al., 1995; Gantois et al., 2009). Therefore, reducing *S*. Enteritidis in the chicken intestinal tract would reduce contamination of poultry products, minimizing human health risk (Altekruse et al., 1993). Thus, intervention strategies for controlling *S*. Enteritidis colonization in chickens are critical for improving the microbiological safety of poultry-derived foods. Since a multitude of sources can transmit *S*. Enteritidis to chickens at farms, a variety of pre-harvest approaches, especially in-feed supplementation of antimicrobials, has been explored for reducing the pathogen persistence in poultry (reviewed in Kollanoor Johny et al., 2012a,b,c).

We previously reported that two phytophenolics, namely *trans*-cinnamaldehyde (TC) and eugenol (EG), were effective in reducing *S*. Enteritidis *in vitro* and in broiler chickens (Kollanoor Johny et al., 2008, 2010, 2012a,b,c). Further, we observed that in-feed supplementation of TC in layers significantly reduced *S*. Enteritidis colonization in the internal organs of birds and egg-borne transmission of the bacterium (Upadhyaya et al., 2015). *Tran*s-cinnamaldehyde, an aromatic aldehyde extracted from the bark of cinnamon (*Cinnamomum zeylandicum*), has well-known antimicrobial properties (Friedman et al., 2002, reviewed by Burt, 2004). Eugenol (EG), a compound obtained from clove (*Eugenia caryophillis*) oil, is also reported to be effective in killing pathogenic microorganisms (Suhr and Nielsen, 2003, reviewed by Burt, 2004). Both these compounds are classified as generally recognized as safe (GRAS) for use in foods by the United States Food and Drug Administration (USFDA) (TC–21CFR182.60; EG–21CFR582.60).

In light of our previous findings, the objective of this study was to analyze the genome-wide response of *S*. Enteritidis Phage Type 8 (or PT8) to a sub-inhibitory concentration of TC or EG using DNA microarrays. We sought to delineate the arsenal of genes affected by the two phytochemicals.

## Results

The DNA microarray analysis revealed that about 10% of genes in the *S*. Enteritidis PT8 genome were significantly modulated after exposure to TC and EG. The major genes predicted to be differentially regulated in PT8 exposed to TC and EG based on the microarray data are provided in Table 1. The analysis identified 566 genes (9.7% genes on the array) with differentially expressed transcripts due to exposure to TC, and 483 genes (8.4% on the array) with differential expression as a result of EG exposure (M ± 1.5, *P* < 0.005; Tables 2, 3). TC significantly down-regulated 275 genes, whereas EG did so for 289 genes (*P* < 0.005). Many of the genes modulated are known to play a critical role in *S*. Enteritidis virulence *in vivo* based on previously published reports (Dhawi et al., 2011; Harvey et al., 2011) and are discussed here. Additional data on genes that are not discussed in depth in the manuscript can be found in the Supplementary Tables S1–S19.

**Table 1:** Major genes predicted to be differentially regulated in PT8 exposed to SICs of both TC and EG (*P* < 0.005, M ± 1.5), based on the microarray data.

**Gene**	**Definition/Function/Gene product**	**TC**	**EG**
***Salmonella*** **PATHOGENICITY ISLAND-1 (SPI-1)**			
*hilC*	Invasion genes transcription activator	−2.2	−3.0
*hilD*	Regulatory helix-turn-helix proteins, araC family	−2.0	−1.6
*sicA*	Surface presentation of antigens; secretory proteins	−1.4	−1.5
*sprB*	Possible AraC-family transcriptional regulator	−1.6	−1.5
**MOTILITY AND CHEMOTAXIS**			
*flhD*	Regulator of flagellar biosynthesis, acts on class 2 operons	−1.5	−1.5
*fliC*	Flagellar biosynthesis; flagellin, filament structural protein	−1.4	−2.2
*fliZ*	Putative regulator of FliA	−1.3	−2.4
**OUTER MEMBRANE PROTEINS**			
*ompW*	Outer membrane protein W; colicin S4 receptor	−4.3	−2.7
*ompC*	Outer membrane protein 1b (ib;c), porin	−2.8	−2.8
*ompS1*	Putative porin	−2.3	−1.7
*nmpC*	New outer membrane protein; predicted bacterial porin	−4.6	−3.5
*tsx*	Nucleoside channel receptor of phage T6 and colicin K	−1.8	−1.8
**CARBOHYDRATE METABOLISM AND TRANSPORT**			
*agp*	Glucose-1-phosphatase	−1.8	−1.8
*mglB*	ABC superfamily (peri_perm), galactose transport protein	−1.9	−3.4
*cydA*	Cytochrome d terminal oxidase, polypeptide subunit I	−3.1	−3.0
*frdA*	Fumarate reductase, anaerobic, flavoprotein subunit	−2.0	−2.6
*frdB*	fumarate reductase, anaerobic, Fe-S protein subunit	−2.0	−2.0
*mglA*	ABC superfamily, galactose transport protein	−1.7	−2.8
*mglC*	ABC superfamily, methyl-galactoside transport protein	−1.5	−1.8
**AMINOACID TRANSPORT**			
*tdcB*	Threonine dehydratase, catabolic	−2.4	−3.7
*tdcA*	Transcriptional activator of tdc operon (LysR family)	−2.2	−3.0
*tdcG*	L-serine deaminase	−2.8	−3.0
*pepT*	Putative peptidase T (aminotripeptidase)	−2.5	−1.6
*speF*	Ornithine decarboxylase isozyme, inducible	−5.2	−4.3
*potE*	APC family, putrescine/ornithine antiporter	−4.2	−3.2
**TETRATHIONATE REDUCTION**			
*ttrS*	Tetrathionate reductase complex: sensory kinase	−2.2	−2.3
**PROPANEDIOL UTILIZATION**			
*pocR*	Propanediol utilization: transcriptional regulation, AraC family	−2.5	−2.2
*pduA*	Propanediol utilization: polyhedral bodies	−2.5	−1.9
*pduB*	Propanediol utilization: polyhedral bodies	−4.0	−3.5
*pduC*	Propanediol utilization: dehydratase, large subunit	−4.2	−3.8
*pduD*	Propanediol utilization: dehydratase, medium subunit	−4.2	−4.6
*pduE*	Propanediol utilization: dehydratase, small subunit	−2.1	−1.6
*pduG*	Propanediol utilization: diol dehydratase reactivation	−3.2	−3.0
*pduK*	Propanediol utilization: polyhedral bodies	−2.5	−2.6
*pduL*	Propanediol utilization	−2.5	−3.3
*pduM*	Propanediol utilization	−2.4	−2.3
*pduO*	Propanediol utilization: B12 related	−1.9	−1.8
*pduP*	Propanediol utilization: CoA-dependent dehydrogenase	−2.6	−3.1
*pduQ*	Propanediol utilization: propanol dehydrogenase	−1.8	−2.6
*pduS*	Propanediol utilization: polyhedral bodies	−1.7	−2.0
*pduT*	Propanediol utilization: polyhedral bodies	−2.0	−2.6
*pduU*	Propanediol utilization: polyhedral bodies	−1.6	−2.5
*pduW*	Propanediol utilization: propionate kinase	−1.9	−3.1
**ETHANOLAMINE UTILIZATION**			
*eutE*	Putative aldehyde oxidoreductase in ethanolamine utilization	−2.6	−1.6
*eutT*	Putative cobalamin adenosyltransferase, ethanolamine utilization	−2.8	−1.6
*eutQ*	Putative ethanolamine utilization protein	−3.1	−1.9
*eutS*	Putative carboxysome structural protein, ethanol utilization	−1.5	−1.6
**VITAMIN B12 SYNTHESIS**			
*cbiP*	Putative carboxysome structural protein, ethanol utilization	−1.8	−2.0
*cboQ*	Synthesis of vitamin B12 adenosyl cobalamide precursor	−2.2	−2.6
*cbiN*	Synthesis of vitamin B12 adenosyl cobalamide precursor	−1.7	−2.1
*cbiM*	Synthesis of vitamin B12 adenosyl cobalamide precursor	−2.8	−2.6
*cbiL*	Synthesis of vitamin B12 adenosyl cobalamide precursor	−2.7	−2.6
*cbiH*	Synthesis of vitamin B12 adenosyl cobalamide precursor	−2.0	−2.4
*cbiG*	Synthesis of vitamin B12 adenosyl cobalamide precursor	−2.3	−2.5
*cbiF*	Synthesis of vitamin B12 adenosyl cobalamide precursor	−2.3	−2.4
*cbiC*	Synthesis of vitamin B12 adenosyl cobalamide precursor	−1.7	−1.8
*cbiA*	Synthesis of vitamin B12 adenosyl cobalamide precursor	−2.2	−1.8
**HEAT SHOCK**			
*dnaK*	Chaperone Hsp70 in DNA biosynthesis/cell division	2.9	3.3
*dnaJ*	Heat shock protein, DnaJ and GrpE stimulates DnaK	2.7	3.3
*ibpA*	Small heat shock protein	4.1	4.3
*ibpB*	Small heat shock protein	5.8	3.5
*mopA*	Chaperone Hsp60 with peptide-dependent ATPase activity	2.8	3.3
*mopB*	Chaperone Hsp10, affects cell division	1.7	2.3
*htpG*	Chaperone Hsp9	3.2	4.0
**ANTIBIOTIC RESISTANCE**			
*marA*	Transcriptional Activator	5.5	4.5
*marR*	Transcriptional repressor of *marRAB* operon	3.7	5.9

**Table 2:** Select genes predicted to be differentially regulated in PT8 exposed to SICs of TC but not EG (*P* < 0.005, M ± 1.5), based on the microarray data.

**Gene**	**Function**	***M*-value**
**INVASION**		
*sipA*	Cell invasion protein	−1.8
*sipD*	Cell invasion protein	−1.8
*sipC*	Cell invasion protein	−1.8
*sipB*	Cell invasion protein	−2.1
*sopB*	*Salmonella* outer protein: homologous to *ipgD* of *Shigella*	−1.9
**TETRATHIONATE, ETHANOLAMINE, AND DIMETHYLSUPHIDE REDUCTION**		
*pduF*	Propanediol utilization: propanediol diffusion facilitator	−2.4
*pduN*	Propanediol utilization: polyhedral bodies	−1.6
*eutC*	Ethanolamine ammonia-lyase, light chain	−1.5
*eutB*	Ethanolamine ammonia-lyase, heavy chain	−2.1
*eutH*	Putative transport protein, ethanolamine utilization	−2.1
*eutJ*	Paral Putative heatshock protein (Hsp70)	−2.1
*eutN*	Putative detox protein in ethanolamine utilization	−2.3
*dmsB*	Anaerobic dimethyl sulfoxide reductase subunit B	−4.5
*dmsA*	Putative anaerobic dimethyl sulfoxide reductase, subunit A pseudogene	−5.3
*dmsC*	Putative dimethyl sulfoxide reductase subunit C	−3.0
*STM1498*	Putative dimethyl sulfoxide reductase	−2.9
*STM1499*	Putative dimethyl sulfoxide reductase, chain A1	−2.9
*dmsB*	Anaerobic dimethyl sulfoxide reductase subunit B	−4.5
*dmsA*	Putative anaerobic dimethyl sulfoxide reductase, subunit A pseudogene	−5.3
**HYDROGEN SULFIDE PRODUCTION**		
*phsC*	Hydrogen sulfide production: membrane anchoring protein	−2.6
*phsB*	Hydrogen sulfide production: iron- sulfur subunit; electron transfer	−3.0
*phsA*	Hydrogen sulfide production: membrane anchoring protein	−2.8
*phsC*	Hydrogen sulfide production: membrane anchoring protein	−2.6

**Table 3:** Select genes predicted to be differentially regulated in PT8 exposed to SICs of EG, but not TC (*P* < 0.005, M ± 1.5), based on the microarray data.

**Gene**	**Function**	***M*-value**
**MOTILITY**		
*flhC*	Flagellar transcriptional activator	−2.5
*motA*	Proton conductor component of motor, torque generator	−3.3
*motB*	Enables flagellar motor rotation, linking torque machinery to cell wall	−3.8
*cheA*	Sensory histidine protein kinase, transduces signal between chemo- signal receptors and CheB and CheY	−4.4
*cheB*	cheA sensor	−2.8
*cheM*	Chemotaxis protein II	−4.2
*cheR*	Response regulator for chemotaxis	−2.7
*cheW*	Purine-binding chemotaxis protein; regulation	−1.6
*cheY*	Chemotaxis regulator, transmits chemoreceptor signals to flagellar motor components	−2.8
*cheZ*	Chemotactic response; CheY protein phophatase	−2.4
*fliA*	Sigma F (sigma 28) factor of RNA polymerase, transcription of late flagellar genes (class 3a and 3b operons)	−3.2
*fliD*	Flagellar biosynthesis; filament capping protein; enables filament assembly	−1.9
*fliC*	Flagellar biosynthesis; flagellin, filament structural protein	−2.2
*fliT*	Flagellar biosynthesis; possible export chaperone for FliD	−2.9
*flgK*	Flagellar biosynthesis, hook-filament junction protein 1	−4.4
*flgL*	Flagellar biosynthesis; hook-filament junction protein	−3.5
*flgM*	Anti-FliA (anti-sigma) factor; also known as RflB protein	−3.2
*flgN*	Flagellar biosynthesis: belived to be export chaperone for FlgK and FlgL	−1.7
**TETRATHIONATE AND DIMETHYLSUPHIDE REDUCTION**		
*ttrA*	Tetrathionate reductase complex, subunit A	−2.1
*ttrB*	Tetrathionate reductase complex, subunit B	−1.6
*ttrR*	Tetrathionate reductase complex: response regulator	−2.0
*dmsB*	Anaerobic dimethyl sulfoxide reductase subunit B	−3.2
*dmsA*	Putative anaerobic dimethyl sulfoxide reductase, subunit A pseudogene	−3.9
*dmsC*	Putative dimethyl sulfoxide reductase subunit C	−1.5
*STM1498*	Putative dimethyl sulfoxide reductase	−2.1
**HYDROGEN SULPHIDE PRODUCTION**		
*phsC*	Hydrogen sulfide production: membrane anchoring protein	−2.7
*phsB*	Hydrogen sulfide production: iron- sulfur subunit; electron transfer	−2.6
*phsA*	Hydrogen sulfide production: membrane anchoring protein	−3.0

### *Salmonella* pathogenicity Island-1 (SPI-1) and type three secretion system genes (T3SS)

The virulence genes associated with the major pathogenicity island, SPI-1, were affected in response to TC exposure, including major regulators, such as *hilC* and *hilD* (Table 1). Transcription of these genes was down-regulated 2.0- and 2.2-fold, respectively, by TC (*P* < 0.005). In addition, genes associated with cell invasion (*sipABCD*) and outer membrane protein syntheses (*sopB*) were significantly down-regulated by TC (Table 2).

### Motility, chemotaxis, and adherence

Several genes associated with *Salmonella* motility, chemotaxis and adherence to host cells were down-regulated by both plant-derived compounds, although EG was significantly more effective than TC (Tables 1, 3). For example, EG significantly down-regulated the transcription of genes responsible for bacterial motility, including *motA, motB*, and *flhC*, chemotaxis genes, *cheA, cheY* and *cheZ*, and the major flagellin *fliC* (Table 3).

### Outer membrane proteins (OMPs)

Both plant-derived compounds significantly down-regulated genes encoding OMPs, namely *ompW, ompC, ompS1*, and *nmpC* (Table 1). The *nmpC* gene, encoding a predicted bacterial porin, was down-regulated by 4.6- and 3.5-fold by TC and EG, respectively. Similarly, *ompW*, a colicin S4 receptor gene, was also reduced in transcription by 4.3- and 2.7-fold, respectively, by TC and EG. However, some of the major OMP genes, such as *ompA, ompR*, and *ompX*, were not affected by both compounds.

### Metabolism and biosynthetic pathway genes

As revealed by microarray analysis, several genes responsible for energy production and conversion in *Salmonella* were down-regulated by TC and EG. For example, the majority of the genes regulating the carbohydrate metabolism and transport, amino acid transport, and carbon compound degradation (Table 1, Supplementary Table S11) were found to be down-regulated by TC and EG. There were indications of down-regulation of mixed metabolism genes, including genes related to the glucose metabolism and genes associated with C4- (e.g., fumarate), non-glucose C6- (e.g., gluconate, fucose), and C9- (sialic acid) carbohydrate metabolisms. In addition, *melA, melB*, and *melR*, genes regulated by the melibiose operon, and mannose-specific PTS (phosphotransferase) family genes *manXYZ* were down-regulated by both plant-derived compounds (Table 1). A complete list of these genes is found in Supplementary Tables S8–S18.

A major set of genes involved in tetrathionate reduction (*ttr*), propanediol utilization (*pdu*), ethanolamine utilization (*eut*), and dimethyl sulfide reduction (*dms*) were down regulated by TC and EG (Tables 1–3). Moreover, the *cbi* locus genes associated with vitamin B12 synthesis (Table 1) were down-regulated by TC and EG. Furthermore, genes responsible for H_2_S production, *phsABC*, were significantly down-regulated by both compounds (Tables 2, 3).

### Up-regulated genes

Besides the down-regulation of several genes indicated above, a few critical genes were up-regulated in response to TC and EG exposure (Table 1, Supplementary Table S19). The greatest increase in transcription was observed with heat shock proteins. The genes encoding two small heat shock proteins, *ibpA* and *ibpB*, were up-regulated by TC by 4.1- and 5.8-fold, respectively, and 4.3- and 3.5-fold, respectively, by EG. In addition, *dnaJ*, and the Hsp70 chaperone gene *dnaK* were up-regulated on exposure to both compounds. The microarray analysis revealed increased transcription of *acrA* and *acrB*, the acridine efflux pump genes, by EG (Table 1). Similarly, multiple antibiotic resistance protein (*mar*) genes were also up-regulated by TC and EG. However, *marR*, encoding the transcriptional repressor of the *marRAB* operon, was also found to be up-regulated by both plant -derived compounds (*P* < 0.005), probably as a compensatory mechanism.

### Confirmation with RT-qPCR

**Figure 1:**
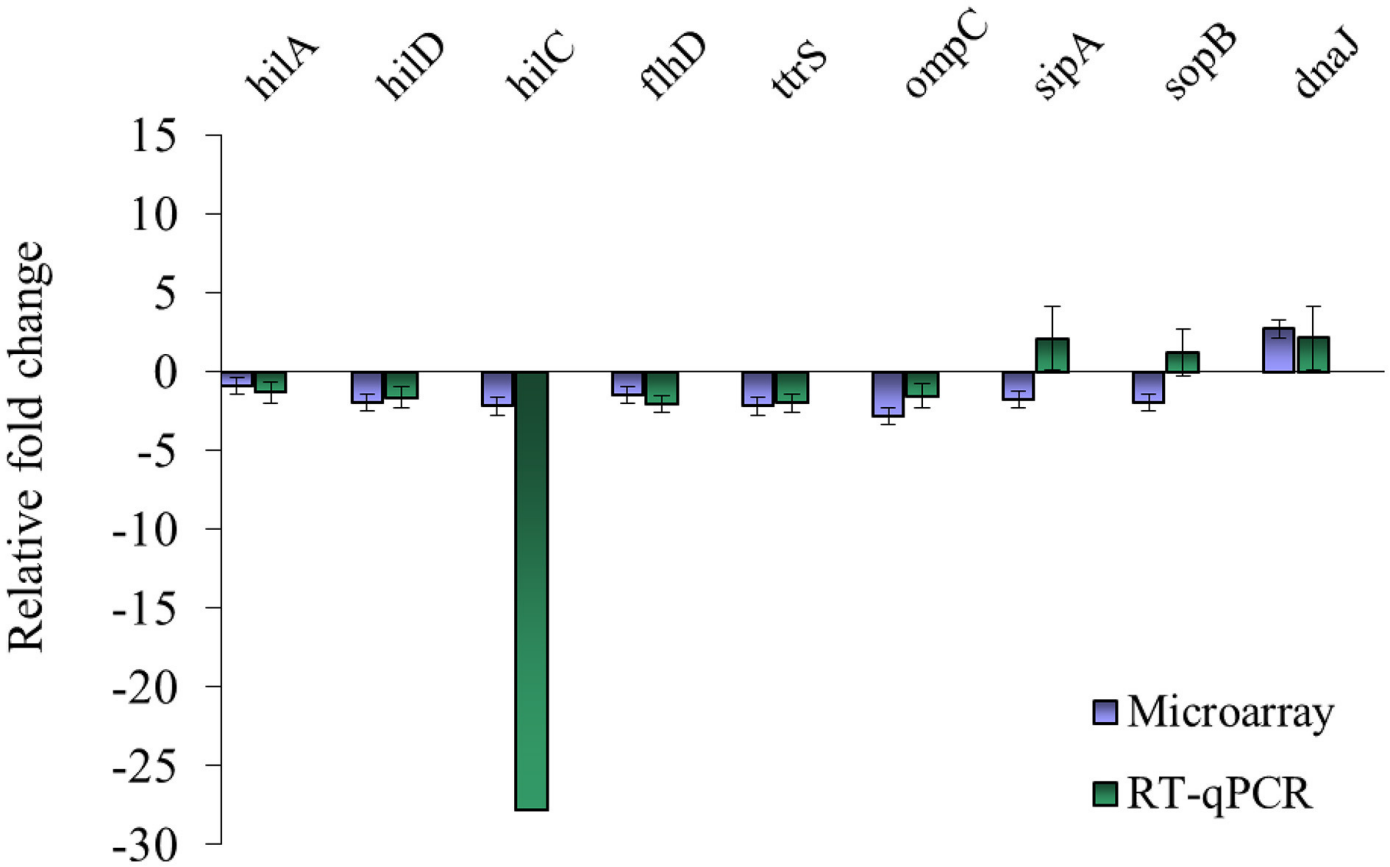
Comparison of microarray and RT-qPCR data on select genes after exposure of *S*. Enteritidis PT8 to SICs of TC. Data are from three replicates.

**Figure 2:**
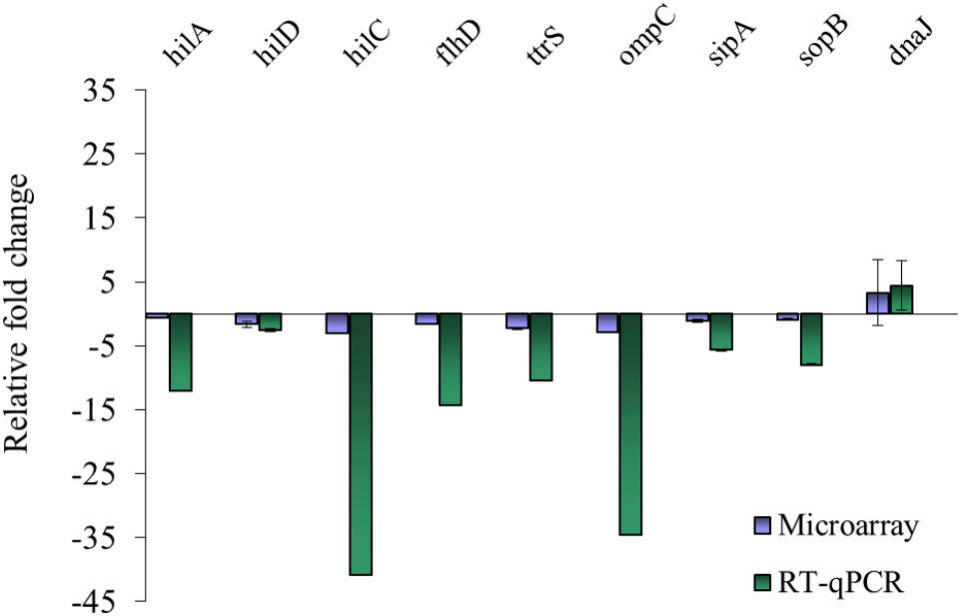
Comparison of microarray and RT-qPCR data on select genes after exposure of *S*. Enteritidis PT8 to SICs of EG. Data are from three replicates.

Nine genes with significantly different transcription levels following TC or EG exposure as identified by microarray analysis were further analyzed by RT-qPCR. Regarding exposure to TC, microarray and RT-qPCR results were in agreement for all tested genes but *sipA* and *sopB* (Figure 1). Microarray data revealed that *sipA* and *sopB* were down-regulated by 1.8- and 2.0-fold, respectively, but in RT-qPCR analysis, a slight up-regulation of both genes on exposure to TC was observed. Regarding exposure to EG, *hilA, hilD, flhD, ttrS, ompC, sipA*, and *sopB* were found significantly down-regulated in microarray analysis and in RT-qPCR results (Figure 2).

## Discussion

*Salmonella* Enteritidis is a leading cause of foodborne salmonellosis worldwide. The increase in the number of outbreaks over the last few decades, where poultry and poultry products were implicated, has led to several pre- and post-harvest strategies to control the pathogen. We previously reported that supplementation of TC and EG in broilers (Kollanoor Johny et al., 2012a,b) and TC in layers (Upadhyaya et al., 2015) significantly reduced *S*. Enteritidis colonization in birds. We also observed that these compounds decreased *Salmonella* motility and invasion of avian epithelial cells *in vitro* (Kollanoor Johny et al., 2012b). Since analysis of transcriptional profiles of bacteria exposed to an antimicrobial molecule can yield new information regarding affected pathways and mechanisms of inhibition (Wilson et al., 1999; Gmuende et al., 2001), we conducted a microarray analysis of *S*. Enteritidis PT8 exposed to TC and EG. The goal was to delineate the genes affected by these plant-derived compounds, to gain insight into the molecular mechanism of how these compounds may cause reduced *S*. Enteritidis colonization in chickens.

A diverse repertoire of *Salmonella* virulence genes was down-regulated in response to treatment with TC and EG, including, critically, the genes involved in SPI-1 regulation. These genes are necessary for *Salmonella* uptake into non-phagocytic epithelial cells (McCormick, 2004). A direct control of SPI-1 genes is exerted by the activation by *hilA*-the major regulator of the island, resulting in the invasion of host tissue. Ellermeier and Slauch (2007) reported that other regulators, such as HilC and HilD work in conjunction with another regulator RtsA to express SPI-1. HilD, an AraC/XylS family member, binds directly to several sites within the *hilA* promoter and derepresses gene expression, which in turn results in the expression of other associated virulence genes. Both plant-derived compounds down-regulated the major regulators *hilA, hilD*, and *hilC*, although a greater effect was observed on *hilC* (Table 1, Supplementary Table S1). The T3SS encoded by SPI-1 is the pathogenic machinery to inject secretory proteins into host cells that help in the internalization of *Salmonella* (Kubori et al., 2000; Galan, 2001). The effector proteins are transported into the host cells with the help of three translocation proteins, *sipB, sipC* and *sipD* (Collazo and Galan, 1997; Galan, 2001). As revealed in the microarray data, TC down-regulated the effector protein genes (Supplementary Table S3).

Flagellar motility and chemotaxis are the two critical features by which bacteria survive under a wide variety of environments (Soutourina and Bertin, 2003), including the intestinal lumen and ceca. Flagellar motility is also required for colonization by *Salmonella* (Ciacci-Woolwine et al., 1998), and is considered a virulence factor in the bacterium (Josenhans and Suerbaum, 2002). In the regulation of flagella, the *flhDC* master operon plays a key role (Kutsukake et al., 1990; Soutourina and Bertin, 2003). Among other genes, the master operon is necessary for transcriptional activation of the *fliA* gene, encoding σ^28^, a sigma factor associated with the transcription of late flagellar genes, such as *fliC* (filament structural protein). It was interesting to note that that these associated genes were all down-regulated by EG (Table 1). Apart from the *flhCD-*regulated genes (early flagellar operon), the middle and late operon genes, such as *flgKLMN, fliZ, motAB*, and *cheAWYZ*, were also down-regulated by EG (Table 3, Supplementary Table S5). In contrast, TC did not exert strong effects on these genes.

Porins are outer membrane aqueous channels used in the diffusion of various compounds across the membrane that can also aid in adapting the bacteria to various conditions and confer antibacterial drug resistance (Gil et al., 2009). In the current study, TC and EG down-regulated porins, such as *ompW, ompC, ompS1*, and *nmpC* by 2-fold. Recently, Gil et al. (2009) reported that *ompW* expression in *Salmonella* was activated in response to oxidative stress. Both plant-derived compounds down-regulated *ompW* by 4.3- and 2.7-fold, respectively. Methner et al. (2004) reported that *ompC* deletion mutants of *S*. Enteritidis resulted in decreased virulence and colonization in chickens in conjunction with deletions of *rpoS* or *phoP*. Our results revealed that *ompC* was down-regulated by 2.8-fold by both compounds (Table 1). Porin *ompS1*, down-regulated by TC and EG in our study, is regulated by the EnvZ/OmpR two-component signal transduction system (Florez-Valdez et al., 2003). Rodriguez-Morales et al. (2006) reported that *ompS1* is required for virulence in *S*. Typhimurium in mice.

It was recently reported that during colonization in the gut *Salmonella* makes use of a wide range of carbon compounds, including D- glucose, melibiose, L-ascorbate, and other unusual compounds, such as sialic acid, ethanolamine, and propanediol resulting from the degradation of host mucosa (Dhawi et al., 2011). We observed that TC and EG down-regulated some of the glucose metabolism genes (*agp, pykA*), and TC reduced the transcription of *melABR* genes (Supplementary Table S8). Moreover, both plant-derived compounds were found to apparently reduce the bacterial capacity to utilize propanediol and ethanolamine as an energy source or tetrathionate as a terminal electron acceptor (Table 1), suggesting that TC and EG may interrupt the pathways critical for *Salmonella* survival and successful competition with the host microflora. During colonization of the host intestine, *Salmonella* can use energy and nutrient sources unavailable to commensal bacteria, including propanediol and ethanolamine. These compounds cannot be used for energy without a terminal electron acceptor, and the host flora mainly consists of mostly fermentative bacteria unable to perform this electron transport. *Salmonella*, however, is uniquely capable of using these compounds by employing tetrathionate as a terminal electron acceptor. When tetrathionate is used as an electron acceptor, it is reduced to thiosulfate, which is then regenerated via oxidation by the reactive oxygen compounds released by the host in response to invasion by *Salmonella*. *Salmonella* can therefore outcompete the host flora, resulting in inflammatory diarrhea, with *Salmonella* cells as major bacterial component of the excreta thus accomplishing transmission to the next host organism.

Harvey et al. (2011) reported that within the lumen of chicks, the degradation of 1, 2-propanediol occurs at a much higher rate, requiring cobalamin synthesis. The *pdu* operon required for propanediol utilization works in conjunction with *cob* and *cbi* operons involved in vitamin B12 synthesis. Our results revealed that TC and EG significantly down-regulated *pdu* and *cbi* operons (Table 1). It was previously observed that *Salmonella* could utilize ethanolamine, which is derived from host cells and membranes, as a sole source of carbon, nitrogen, and energy (Garsin, 2010). Catabolism of ethanolamine is under the control of the *eut* operon that primarily consists of 17 clustered genes (Kofoid et al., 1999) involved in the conversion of the compound to acetyl-CoA for energy (Harvey et al., 2011). The plant compound TC was found to down-regulate these genes (Supplementary Table S14). An anaerobic environment may exist in the chicken cecum (Harvey et al., 2011), and oxygen may not be present as a terminal electron acceptor. In that case, other electron acceptors are required, and ethanolamine and 1, 2-propanediol could be utilized in that function by *Salmonella* in the cecum (Price-Carter et al., 2001). Tetrathionate may be present in the cecum of chicks due to the presence of yolk sac, which is rich in sulfur. Tetrathionate is reduced to thiosulphide and H_2_S, involving proteins encoded by *ttr* and *phs* (Harvey et al., 2011). Our results indicated that TC down-regulated the transcription of *ttr* genes (Supplementary Table S12), whereas both plant-derived compounds reduced *phsABC* transcription (Supplementary Table S18). Overall, TC and EG may disrupt *Salmonella*'s ability to use a unique energy source, possibly reducing its ability to compete successfully with the host's microflora.

Some heat shock genes and genes conferring antibiotic resistance were up-regulated by TC and EG. While the *mar* (multiple antibiotic resistance) locus genes were up-regulated by both TC and EG, the efflux pump genes *acrAB* were only up-regulated by EG. The MarA protein positively regulates transcription of *acrAB*, and it was therefore unexpected that the latter genes were not up-regulated after exposure to TC. However, the transcriptional repressor *marR* was also found to be up-regulated by both TC and EG, potentially indicating a compensatory mechanism on reducing *marAB*-related resistance. Heat shock proteins *dnaJ, dnaK, ibpA, ibpB*, and *htpG* were found to be up-regulated by both plant-derived compounds (*P* < 0.005). However, *clpPX*-mediated heat shock protection mechanisms were least affected (Supplementary Table S19).

Some limitations of this study include the use of nutrient broth for the gene expression studies as opposed to growth within a characterized chicken-derived intestinal cell line, although broth cultures have been previously used for similar experiments (Cao et al., 2011; Jessica et al., 2013; Upadhyay et al., 2013). To our knowledge, immortalized chicken intestinal cell lines are unavailable, and developing and maintaining a primary chicken intestinal cell line has its challenges, including the high variability in confluency across different propagations. In a separate study, we conducted invasion assays using the only available permanent avian cancer cell line (Budgerigar Abdominal Tumor Cells; BATCs) and found that these two phytophenolics significantly reduced *Salmonella* invasion without affecting the viability of cells (Kollanoor Johny et al., 2012b). However, being a permanent cell line obtained from a different avian species (parakeets), a direct correlation of these results to a chicken-derived cell environment is not guaranteed. However, the selected phytophenolics reduced the cecal colonization of *S*. Enteritidis in broiler chickens in at least 5 independent *in vivo* experiments (Kollanoor Johny et al., 2012a,b), lending support to the notion that TC and EG directly affect *S*. Enteritidis virulence genes. However, this hypothesis needs to be further validated.

## Conclusions

Genes required for *S*. Enteritidis PT8 virulence and colonization, including those conferring flagella-associated motility, those enabling invasion of epithelial cells, genes encoding T3SSs, genes regulating the synthesis of effector proteins delivered by T3SS, and genes encoding other surface virulence structures and OMPs were found to be down-regulated after exposure to subinhibitory concentrations of TC or EG. Moreover, the exposure to both phytochemicals brought about transcriptional changes of *S*. Enteritidis PT8 genes involved in the degradation of carbon compounds, those related to amino acid-, carbohydrate-, and lipid-transport, those associated with secondary metabolism, and genes encoding the biosynthesis of molybdopterin and vitamin B12. The genes regulating pathways associated with tetrathionate reduction, propanediol, and ethanolamine utilization, and H_2_S production were also affected. Finally, a few genes regulating antibiotic resistance and heat shock were up-regulated by the plant-derived compounds, including transcriptional activators and repressors, with as yet undefined phenotypic net results.

## Methods

### Bacterial strain and growth conditions

*S*. Enteritidis PT8, obtained from the Connecticut Veterinary Diagnostic Medical Laboratory, was used for the experiments. This strain was used along with three other strains in our *in vitro* invasion and motility assays, and *in vivo* chicken experiments (Kollanoor Johny et al., 2008, 2010, 2012a,b,c). The strain was selected based on RT-qPCR analysis of virulence gene transcription (*hilA, hilD, motA, flhC*, and *invF*) of different phage types (PT-8, -13, and -13b) in response to subinhibitory concentrations of TC and EG (Kollanoor Johny et al., 2012b).

PT8 was grown overnight in Luria-Bertani (LB) broth (Difco) at 37°C with shaking at 150 rpm. For the transcription profiling experiments, 10 ml of an overnight culture was spun at 3,800 × *g* for 15 min, and the resulting pellet was resuspended in 10 ml phosphate buffered saline (PBS; pH 7.0). A 0.5 ml aliquot of the suspension was added to 500 ml of LB broth and grown with vigorous shaking at 37°C until it reached a late-log phase OD_600_ of 0.5. Assays were performed using 3 independent biological replicates for TC and EG, separately. The SIC of TC (0.01% vol/vol; 0.75 mM) or EG (0.04% vol/vol; 2.46 mM) (Kollanoor Johny et al., 2012b) was added to the culture and the mixture was vortexed for 1 min. The control flask was vortexed for 1 min as well. Exactly 100 ml were drawn from the broth before addition of TC or EG (time 0), and 30 min (time 30) after addition of the plant-derived compounds. The samples were drawn separately into 250 ml centrifuge tubes containing 16 ml of ice-cold ethanol/phenol stop solution (5% citrate-buffered phenol v/v), to halt the degradation of mRNA. The cells were spun at 8,000 × *g* for 2 min at 4°C, and the resulting pellet was stored at −80°C until use.

### Total RNA extraction

Total RNA from *S*. Enteritidis PT8 was extracted as previously described (Frye et al., 2006). The frozen pellet was lysed by resuspending in a final volume of a fresh solution of 10 ml 0.5 mg/ml lysozyme in TE (pH 8.0). A volume of 1 ml of 10% SDS was added, and the mixture was placed in a water bath at 64°C for 2 min. After incubation, 11 ml of 1 M sodium acetate solution (pH 5.2) was added to the tubes and mixed. An equal volume of phenol was added and mixed, followed by incubation at 64°C for 6 min. The tubes were then placed on ice to chill before they were spun at 10,000 × *g* for 10 min at 4°C. The aqueous layer was transferred to a fresh tube containing an equal volume of chloroform, mixed, and spun at 10,000 rpm for 5 min at 4°C. The aqueous layer from the samples was then transferred into fresh tubes, 1/10 volume of 3 M sodium acetate (pH 5.2) was added, and one volume of cold isopropanol. The tubes were incubated at −80°C for 20 min before they were spun at 14,000 × *g* for 25 min at 4°C. Isopropanol was carefully removed and the resulting pellet was washed with 40 ml 80% cold ethanol. The pellets were spun at 14,000 × *g* for 5 min at 4°C. Ethanol was carefully removed and the pellet was air dried. The pellet was resuspended in 2 ml of RNase-free DEPC-treated water. The sample was split into two and each was provided with 500 U of RNase inhibitor (Ribolock®, Fermentas), 250 U of DNase (Fermentas), 20 μl of 1 M Tris (pH 8.3) and 10 μl of 1 M MgCl, followed by mixing and incubation at 37°C for 30 min. The RNA sample was then extracted once each with phenol and phenol–chloroform and twice with chloroform. A 1/10th volume of 3 M sodium acetate at pH 5.2 was added, and the RNA was precipitated with isopropanol, washed with 80% ethanol and resuspended in nuclease-free water (Frye et al., 2006). The RNA concentration and purity were determined using a Nanodrop (Thermofisher Corp.).

### Microarray, target preparation, and hybridization

The *Salmonella* whole genome microarray used in this study has been previously described (Porwollik et al., 2003) and includes 5,776 PCR products representing more than 99% of the ORFs of *S*. Enteritidis PT4 as well as four other serotypes of *Salmonella*. The microarray hybridization and analysis were done by standard methods, as previously described for *Salmonella* (Porwollik et al., 2003; Frye et al., 2006). Fifty microgram of RNA was transcribed into cDNA and labeled with Cy3- or Cy5-conjugated dUTP using reverse transcriptase (Superscript II®; Invitrogen) and random hexamers as primers. Unincorporated nucleotides were removed using a PCR purification kit (Qiagen) according to the manufacturer's instructions. Equal volumes of labeled probes from 0 (Cy3-labeled control) and 30 min (Cy5-labeled treatment) were mixed with an equal volume of hybridization solution (50% formamide, 10 × SSC and 0.2% SDS). Slides were prehybridized in 25% formamide, 5 × SSC and 0.1% SDS at 42°C. Probes were hybridized simultaneously to a chip containing three replicate arrays spotted onto CMT-UltraGAPS® (Corning) slides. Slides were washed in stringency-controlled buffers and dried by centrifugation before scanning.

### Scanning and data analysis

The slides were scanned using the ScanArray 5,000 laser scanner (GSI Lumonics). The signals were recorded with scanarray 2.1 software and then quantified using Quantarray 3.0 software (Packard BioScience) (Frye et al., 2006; Ledeboer et al., 2006). Arrays were scanned for high signal intensity and low background in either of the channels, to be included for analysis. The normalization of data considered the median intensity of the spot and the local background. The data were statistically analyzed using the Partek Discovery SuiteTM v 6.2 (Partek Inc., St. Louis, MO). Mixed effects ANOVA was performed, and a significant *P* < 0.005 for each comparison was determined using a false discovery rate of 0.05. A total of 11 (5 treated and 6 untreated) biological replicates (separate RNA preparations) were analyzed from experiments conducted on different days, and the data have been deposited in GEO (https://www.ncbi.nlm.nih.gov/geo/) under accession number GSE102477.

### Real-time quantitative PCR (RT-qPCR)

An RT-qPCR analysis was conducted to confirm the differential expression of the selected microarray targets. The protocol of sample preparation and collection for microarray experiment was also used for preparation of RT-qPCR samples. Total RNA was extracted from three biological replicates of PT8 cultures exposed to TC or EG using the RNeasy minikit (Qiagen) according to manufacturer's instructions. The resulting RNA was treated with RNase-free DNase. The quantitation of RNA was done by measuring the absorbance at 260 and 280 nm (Nanodrop, Biorad). Complementary DNA (cDNA) was synthesized using the Superscript II Reverse transcriptase kit (Superscript™—Invitrogen, Carlsbad, CA). The cDNA was used as the template for the amplification of *Salmonella* genes selected based on the results from microarray, including *hilA, hilD, hilC, flhD, dnaJ, ompC, sipA, sopB*, and *ttrS*. The specific primers for the genes, and for 16S rRNA (endogenous control) (Table 4) were designed using Primer Express software (Applied Biosystems). The primers were synthesized by Integrated DNA Technologies (Foster City, CA). Real time-qPCR was performed with the ABI PRISM 7900 sequence detection system (Applied Biosystems), using the SYBR® Green assay (Applied Biosystems) under custom thermal cycling conditions (denaturation step at 95°C for 10 min, followed by 40 cycles of a denaturation step at 95°C for 15 s, and an annealing/elongation step at 60°C for 60 s). A default melting curve stage (95°C for 15 s, 0°C for 60 s, 95°C for 15 s) was also included. The biological replicates were analyzed in duplicate and normalized against 16S rRNA gene expression. The 16S rRNA gene was used to account for the variability between the samples since the transcript levels of the endogenous control were found to be not significantly different (*P* > 0.05) between the control- and the TC- or EG- treated cells. The comparative Ct method (2^−ΔΔCt^) was used to assess the relative changes in the mRNA expression levels between the control and TC-/EG- treated PT8 (Schmittgen and Livak, 2008).

**Table 4:** Primers used for RT-qPCR assays.

**Gene target**	**Forward primer (5′–3′)**	**Reverse primer (5′−3′)**
*16S rRNA*	CGTGTTGTGAAATGTTGGGTTAA	CCGCTGGCAACAAAGGATAA
*hilA*	TTGCTGACTCAATGCGTTAACA	CATTCTGCCAGCGCACAGTA
*hilD*	CAACGACTTGGCGCTCTCTAT	TCTCTGTGGGTACCGCCATT
*hilC*	CCAGTTTTCGCTTCAGACTTGA	CACCCGCAAATGGTCACA
*flhD*	CGTTTGATCGTCCAGGACAA	TGTTTGCCATCTCTTCGTTGAT
*ttrS*	CAAATATAATTGAGCGTGAGCAACA	TGGTTAATCCAGCACCAGCTAA
*ompC*	ACGCTGCTGCATAAAGTTGTCA	CCGATGTTCTGCCGGAGTT
*sipA*	TCTGCTTTTTTCCCACCATCA	AGATAAACTGCCTGACCCTAAAATTC
*sopB*	GTGCTGCAATAAGTTCGATAAGATTT	ACCGGCCAGCAACAAAAC
*dnaJ*	ATGGTCGTGTGATGCTGAAAGT	CCGCGCATACGGAACAG

## Authors contributions

AK carried out microarray experiments, RT-qPCR assays, involved in the study design and wrote the manuscript. JF trained AK to conduct microarray analysis, preliminary interpretation of statistical analysis and corrected the manuscript. AD and DD participated in the design of the study and corrected the manuscript. SP and MM designed and constructed the microarrays, interpreted the hybridization results and performed statistical analysis of the data. KV conceived and designed the study and corrected the manuscript. All authors read and approved the manuscript.

### Conflict of interest statement

The authors declare that the research was conducted in the absence of any commercial or financial relationships that could be construed as a potential conflict of interest.

## Abbreviations

TC: *trans*-cinnamaldehyde
EG: Eugenol
SIC: subinhibitory concentration
cDNA: complementary DNA
CDC: Centers for Disease Control and Prevention
GRAS: Generally Recognized as Safe
USFDA: United States Food and Drug Administration
RT-qPCR: Real-Time Quantitative Polymerase Chain Reaction
mRNA: messenger RNA
M: Molar.
