# Supplementary material for: Gene Expression Response of Salmonella enterica Serotype Enteritidis Phage Type 8 to Subinhibitory Concentrations of the Plant-Derived Compounds Trans-Cinnamaldehyde and Eugenol
Source: Front Microbiol. 2017 Sep 26;8:1828. doi: 10.3389/fmicb.2017.01828 (PMC5623010; doi:10.3389/fmicb.2017.01828)
Supplement: Supplementary file 1 [file Table1.DOC]

Table S1. Effect of *trans*-cinnamaldehyde and eugenol on expression of SPI-1 genes

| Gene | Function | *Trans*-cinnamaldehyde | | Eugenol | |
| --- | --- | --- | --- | --- | --- |
|  |  | M value | P value | M value | P value |
| *hilC* | Bacterial regulatory helix-turn-helix proteins, araC family | -2.2 | *P* ≤ 0.005 | -3.0 | *P* ≤ 0.005 |
| *hilA* | Invasion genes transcription activator | -0.9 | *P* ≤ 0.005 | -0.6 | *P* ≤ 0.005 |
| *hilD* | Regulatory helix-turn-helix proteins, araC family | -2.0 | *P* ≤ 0.005 | -1.6 | *P* ≤ 0.005 |
| *sicA* | Surface presentation of antigens; secretory proteins | -1.4 | *P* ≤ 0.005 | -1.5 | *P* ≤ 0.005 |
| *prgK* | Cell invasion protein; lipoprotein, may link inner and outer membranes | -0.5 | *P* = 0.02 | -0.6 | *P* ≤ 0.005 |
| *prgH* | Cell invasion protein | -0.9 | *P* ≤ 0.005 | -0.5 | P = 0.01 |
| *invF* | Invasion protein | -0.8 | *P* ≤ 0.005 | -1.0 | *P* ≤ 0.005 |
| *invG* | Invasion protein; outer membrane | -1.0 | *P* ≤ 0.005 | -0.9 | *P* ≤ 0.005 |
| *invJ* | Surface presentation of antigens; secretory proteins | -0.8 | *P* ≤ 0.005 | -0.8 | *P* ≤ 0.005 |
| *orgA* | Putative flagellar biosynthesis/type III secretory pathway protein | -0.8 | *P* ≤ 0.005 | -0.5 | P = 0.02 |
| *sprB* | Possible AraC-family transcriptional regulator | -1.6 | *P* ≤ 0.005 | -1.5 | *P* ≤ 0.005 |
| *sipA* | Cell invasion protein | -1.8 | *P* ≤ 0.005 | -1.1 | *P* ≤ 0.005 |
| *sipD* | Cell invasion protein | -1.8 | *P* ≤ 0.005 | -0.8 | *P* ≤ 0.005 |
| *sipC* | Cell invasion protein | -1.8 | *P* ≤ 0.005 | -0.6 | P = 0.008 |
| *sipB* | Cell invasion protein | -2.1 | *P* ≤ 0.005 | -1.1 | *P* ≤ 0.005 |
| *sopB* | *Salmonella* outer protein: homologous to *ipgD* of *Shigella* | -1.9 | *P* ≤ 0.005 | -0.9 | *P* ≤ 0.005 |

Table S2.Effect of *trans*-cinnamaldehyde and eugenol on expression of *hyb* operon

| Gene | Function | *Trans*-cinnamaldehyde | | Eugenol | |
| --- | --- | --- | --- | --- | --- |
|  |  | M value | P value | M value | P value |
| *hybF* | Putative hydrogenase expression/formation protein | -0.7 | *P* ≤ 0.005 | -0.7 | *P* ≤ 0.005 |
| *hybE* | Putative hydrogenase | -0.8 | *P* ≤ 0.005 | -0.5 | *P* = 0.02 |
| *hybD* | Putative processing element for hydrogenase-2 | -2.2 | *P* ≤ 0.005 | -2.8 | *P* ≤ 0.005 |
| *hybC* | Hydrogenase-2, large subunit | -2.8 | *P* ≤ 0.005 | -2.2 | *P* ≤ 0.005 |
| *hybB* | Putative cytochrome Ni/Fe component of hydrogenase-2 | -3.0 | *P* ≤ 0.005 | -2.6 | *P* ≤ 0.005 |
| *hybA/O* | function unknown, initially thought to be hydrogenase-2 small subunit | -3.0 | *P* ≤ 0.005 | -2.9 | *P* ≤ 0.005 |
| *hypO* | Putative Ni/Fe hydrogenases, small subunit | -2.9 | *P* ≤ 0.005 | -2.5 | *P* ≤ 0.005 |

Table S3. Effect of *trans*-cinnamaldehyde and eugenol on translocation of genes responsible for effector proteins

| Gene | Function | *Trans*-cinnamaldehyde | | Eugenol | |
| --- | --- | --- | --- | --- | --- |
|  |  | M value | P value | M value | P value |
| *sipB* | Pathogenicity island 1 effector protein | -2.2 | *P* ≤ 0.005 | -1.1 | *P* ≤ 0.005 |
| *sipC* | Pathogenicity island 1 effector protein | -1.6 | *P* ≤ 0.005 | -0.7 | *P* ≤ 0.005 |
| *sipD* | Pathogenicity island 1 effector protein | -1.6 | *P* ≤ 0.005 | -0.8 | *P* ≤ 0.005 |

Table S4. Effect of *trans*-cinnamaldehyde and eugenol on genes responsible for proteins delivered by SPI-1

| Gene | Function | *Trans*-cinnamaldehyde | | Eugenol | |
| --- | --- | --- | --- | --- | --- |
|  |  | M value | P Value | M value | P value |
| *sipA* | Pathogenicity island 1 effector protein (function unknown) | -1.8 | *P* ≤ 0.005 | -1.1 | *P* ≤ 0.005 |
| *sipB* | Pathogenicity island 1 effector protein | -2.1 | *P* ≤ 0.005 | -1.1 | *P* ≤ 0.005 |
| *sipC* | Pathogenicity island 1 effector protein | -1.6 | *P* ≤ 0.005 | -0.7 | *P* ≤ 0.005 |
| *sipD* | Pathogenicity island 1 effector protein | -1.6 | *P* ≤ 0.005 | -0.8 | *P* ≤ 0.005 |
| *avrA* | Putative inner membrane protein | -0.8 | *P* ≤ 0.005 | -1.5 | *P* ≤ 0.005 |
| *sopB* | *Salmonella* outer protein: homologous to *ipgD* of *Shigella* | -2.0 | *P* ≤ 0.005 | -0.9 | *P* ≤ 0.005 |
| *sptP* | Protein tyrosine phosphate | -1.5 | *P* ≤ 0.005 | -0.6 | *P* = 0.02 |

Table S5. Effect of *trans*-cinnamaldehyde and eugenol on motility genes

| *Gene* | Function | *Trans*-cinnamaldehyde | | Eugenol | |
| --- | --- | --- | --- | --- | --- |
|  |  | M value | P value | M value | P value |
| *flhD* | Regulator of flagellar biosynthesis, acts on class 2 operons | -1.5 | *P* ≤ 0.005 | -1.5 | *P* ≤ 0.005 |
| *flhC* | Flagellar transcriptional activator | -1.0 | *P* ≤ 0.005 | -2.5 | *P* ≤ 0.005 |
| *motA* | Proton conductor component of motor, torque generator | -0.5 | *P* ≤ 0.005 | -3.3 | *P* ≤ 0.005 |
| *motB* | Enables flagellar motor rotation, linking torque machinery to cell wall | -0.5 | *P* ≤ 0.005 | -3.8 | *P* ≤ 0.005 |
| *cheA* | Sensory histidine protein kinase, transduces signal between chemo- signal receptors and CheB and CheY | -1.0 | *P* ≤ 0.005 | -4.4 | *P* ≤ 0.005 |
| *cheB* | cheA sensor | NS | *P* ≤ 0.005 | -2.8 | *P* ≤ 0.005 |
| *cheM* | Chemotaxis protein II | NS | *P* ≤ 0.005 | -4.2 | *P* ≤ 0.005 |
| *cheR* | Response regulator for chemotaxis | NS | *P* ≤ 0.005 | -2.7 | *P* ≤ 0.005 |
| *cheW* | Purine-binding chemotaxis protein; regulation | -1.0 | *P* ≤ 0.005 | -1.6 | *P* ≤ 0.005 |
| *cheY* | Chemotaxis regulator, transmits chemoreceptor signals to flagellar motor components | -0.4 | *P* ≤ 0.005 | -2.8 | *P* ≤ 0.005 |
| *cheZ* | Chemotactic response; CheY protein phophatase | -0.6 | *P* ≤ 0.005 | -2.4 | *P* ≤ 0.005 |
| *fliC* | Flagellar biosynthesis; flagellin, filament structural protein | -1.4 | *P* ≤ 0.005 | -2.2 | *P* ≤ 0.005 |
| *fliA* | Sigma F (sigma 28) factor of RNA polymerase, transcription of late flagellar genes (class 3a and 3b operons) | -1.0 | *P* ≤ 0.005 | -3.2 | *P* ≤ 0.005 |
| *fliZ* | Putative regulator of FliA | -1.3 | *P* ≤ 0.005 | -2.4 | *P* ≤ 0.005 |
| *fliD* | Flagellar biosynthesis; filament capping protein; enables filament assembly | NS | *P* ≤ 0.005 | -1.9 | *P* ≤ 0.005 |
| *fliC* | Flagellar biosynthesis; flagellin, filament structural protein | NS | *P* ≤ 0.005 | -2.2 | *P* ≤ 0.005 |
| *fliS* | Flagellar biosynthesis; repressor of class 3a and 3b operons (RflA activity) | NS | *P* ≤ 0.005 | NS | *P* ≤ 0.005 |
| *fliT* | Flagellar biosynthesis; possible export chaperone for FliD | NS | *P* ≤ 0.005 | -2.9 | *P* ≤ 0.005 |
| *flgK* | Flagellar biosynthesis, hook-filament junction protein 1 | NS | *P* ≤ 0.005 | -4.4 | *P* ≤ 0.005 |
| *flgL* | Flagellar biosynthesis; hook-filament junction protein | NS | *P* ≤ 0.005 | -3.5 | *P* ≤ 0.005 |
| *flgM* | Anti-FliA (anti-sigma) factor; also known as RflB protein | NS | *P* ≤ 0.005 | -3.2 | *P* ≤ 0.005 |
| *flgN* | Flagellar biosynthesis: belived to be export chaperone for FlgK and FlgL | NS | *P* ≤ 0.005 | -1.7 | *P* ≤ 0.005 |

Table S6. Effect of *trans*-cinnamaldehyde and eugenol on genes responsible for OMPs

| Gene | Function | *Trans*-cinnamaldehyde | | Eugenol | |
| --- | --- | --- | --- | --- | --- |
|  |  | M value | P Value | M value | P value |
| *ompW* | Outer membrane protein W; colicin S4 receptor; Putative transporter | -4.3 | *P* ≤ 0.005 | -2.7 | *P* ≤ 0.005 |
| *ompN* | Outer membrane protein N, non-specific porin | -1.1 | *P* ≤ 0.005 | -0.5 | *P* ≤ 0.05 |
| *ompC* | Outer membrane protein 1b, porin | -2.8 | *P* ≤ 0.005 | -2.8 | *P* ≤ 0.005 |
| *ompR* | Response regulator in two-component regulatory system with EnvZ, affecting transcription of ompC and ompF (OmpR family) | 0.2 | NS | 1.8 | *P* ≤ 0.005 |
| *ompX* | Outer membrane protease, receptor for phage OX2 | 1.7 | *P* ≤ 0.005 | 1.7 | *P* ≤ 0.005 |
| *ompF* | Outer membrane protein 1a, porin | -0.5 | *P* ≤ 0.005 | -0.5 | *P* ≤ 0.005 |
| *ompS1* | Putative porin | -2.3 | *P* ≤ 0.005 | -1.7 | *P* ≤ 0.005 |
| *nmpC* | New outer membrane protein; predicted bacterial porin | -4.6 | *P* ≤ 0.005 | -3.5 | *P* ≤ 0.005 |
| *tsx* | Nucleoside channel receptor of phage T6 and colicin K | -1.8 | *P* ≤ 0.005 | -2.6 | *P* ≤ 0.005 |

Table S7. Effect of *trans*-cinnamaldehyde and eugenol on replication, cell division, transcription and translation genes

| Gene | Function | *Trans*-cinnamaldehyde | | Eugenol | |
| --- | --- | --- | --- | --- | --- |
|  |  | M value | P Value | M value | P value |
| *ftsX* | Putative integral membrane cell division protein | 0.2 | NS | 0.1 | NS |
| *stcC* | Putative outer membrane protein | 0.2 | NS | 0.1 | NS |
| *sbcC* | ATP-dependent dsDNA exonuclease | 0.1 | NS | 0.1 | NS |
| *recD* | Exonuclease V, alpha chain | -0.2 | NS | -0.3 | NS |
| *recB* | Exonuclease V, alpha chain | -0.6 | *P* ≤ 0.005 | -0.4 | NS |
| *recC* | Exonuclease V, beta chain | 0.3 | NS | 0.2 | NS |
| *rmbA* | Putative cytoplasmic protein | 0.2 | NS | 0.3 | NS |
| *fimW* | Putative fimbrial protein | -0.5 | *P* ≤ 0.005 | -0.2 | NS |
| *argS* | Arginine tRNA synthetase | -0.7 | *P* ≤ 0.005 | -0.5 | *P* ≤ 0.05 |
| *nlpL* | Lipoprotein, cell division | -1.5 | *P* ≤ 0.005 | -1.0 | *P* ≤ 0.005 |
| *rpsO* | 30S ribosomal subunit protein S15 | 0.4 | NS | -0.1 | NS |

Table S8. Effect of *trans*-cinnamaldehyde and eugenol on genes responsible for carbohydrate metabolism and transport

| Gene | Function | *Trans*-cinnamaldehyde | | Eugenol | |
| --- | --- | --- | --- | --- | --- |
|  |  | M value | P Value | M value | P value |
| *melR* | Regulator of melibiose operon (AraC/XylS family) | -1.4 | *P* ≤ 0.005 | 0.0 | NS |
| *melA* | Alpha-galactosidase | -2.8 | *P* ≤ 0.005 | -1.1 | *P* ≤ 0.005 |
| *melB* | GPH family, melibiose permease II | -1.5 | *P* ≤ 0.005 | -0.8 | *P* ≤ 0.005 |
| *agp* | glucose-1-phosphatase | -1.8 | *P* ≤ 0.005 | -1.8 | *P* ≤ 0.005 |
| *pykA* | pyruvate kinase II, glucose stimulated | -1.7 | *P* ≤ 0.005 | -1.8 | *P* ≤ 0.005 |
| *mglB* | ABC superfamily (peri_perm), galactose transport protein | -1.9 | *P* ≤ 0.005 | -3.4 | *P* ≤ 0.005 |
| *nagE* | Sugar Specific PTS family, n-acetylglucosamine-specific enzyme IIABC | -1.0 | *P* ≤ 0.005 | -0.5 | *P* ≤ 0.005 |
| *manZ* | Sugar Specific PTS family, mannose-specific enzyme IID | -1.4 | *P* ≤ 0.005 | -0.6 | *P* ≤ 0.005 |
| *manY* | Sugar Specific PTS family, mannose-specific enzyme IIC | -1.6 | *P* ≤ 0.005 | -1.0 | *P* ≤ 0.005 |
| *manX* | Sugar Specific PTS family, mannose-specific enzyme IIAB | -2.0 | *P* ≤ 0.005 | -1.0 | *P* ≤ 0.005 |
| *cydA* | cytochrome d terminal oxidase, polypeptide subunit I | -3.1 | *P* ≤ 0.005 | -3.0 | *P* ≤ 0.005 |
| *cydB* | cytochrome d terminal oxidase | -3.0 | *P* ≤ 0.005 | -2.6 | *P* ≤ 0.005 |
| *frdA* | fumarate reductase, anaerobic, flavoprotein subunit | -2.0 | *P* ≤ 0.005 | -2.6 | *P* ≤ 0.005 |
| *frdB* | fumarate reductase, anaerobic, Fe-S protein subunit | -2.0 | *P* ≤ 0.005 | -2.0 | *P* ≤ 0.005 |
| *frdC* | fumarate reductase, anaerobic, membrane anchor polypeptide | -2.0 | *P* ≤ 0.005 | -1.1 | *P* ≤ 0.005 |
| *frdD* | fumarate reductase, anaerobic, membrane anchor polypeptide | -1.6 | *P* ≤ 0.005 | -1.1 | *P* ≤ 0.005 |
| *mglA* | ABC superfamily (atp_bind), galactose (methyl-galactoside) transport protein | -1.7 | *P* ≤ 0.005 | -2.8 | *P* ≤ 0.005 |
| *mglC* | ABC superfamily (membrane), methyl-galactoside transport protein | -1.5 | *P* ≤ 0.005 | -1.8 | *P* ≤ 0.005 |

Table S9. Effect of *trans*-cinnamaldehyde and eugenol on amino acid transport genes

| Gene | Function | *Trans*-cinnamaldehyde | | Eugenol | |
| --- | --- | --- | --- | --- | --- |
|  |  | M value | P Value | M value | P value |
| *pduU* | Propanediol utilization: polyhedral bodies | -2.5 | *P* ≤ 0.005 | -1.6 | *P* ≤ 0.005 |
| *eutT* | Putative cobalamin adenosyltransferase, ethanolamine utilization | -1.6 | *P* ≤ 0.005 | -2.8 | *P* ≤ 0.005 |
| *eutQ* | Putative ethanolamine utilization protein | -1.9 | *P* ≤ 0.005 | -3.1 | *P* ≤ 0.005 |
| *eutP* | Putative ethanolamine utilization protein | -1.1 | *P* ≤ 0.005 | -2.1 | *P* ≤ 0.005 |
| *eutS* | Putative carboxysome structural protein, ethanol utilization | -1.6 | *P* ≤ 0.005 | -1.5 | *P* ≤ 0.005 |
| *tdcB* | Threonine dehydratase, catabolic | -2.4 | *P* ≤ 0.005 | -3.7 | *P* ≤ 0.005 |
| *tdcG* | L-serine deaminase | -2.8 | *P* ≤ 0.005 | -3.0 | *P* ≤ 0.005 |
| *tdcA* | transcriptional activator of tdc operon (LysR family) | -2.2 | *P* ≤ 0.005 | -3.0 | *P* ≤ 0.005 |
| *tdcC* | HAAAP family, L-threonine/ L-serine permease, anaerobically inducible | -2.4 | *P* ≤ 0.005 | -2.7 | *P* ≤ 0.005 |
| *tdcD* | propionate kinase/acetate kinase II, anaerobic | -2.6 | *P* ≤ 0.005 | -3.2 | *P* ≤ 0.005 |
| *tdcE* | pyruvate formate-lyase 4/ 2-ketobutyrate formate-lyase | -2.5 | *P* ≤ 0.005 | -2.6 | *P* ≤ 0.005 |
| *pepT* | putative peptidase T(aminotripeptidase) | -2.5 | *P* ≤ 0.005 | -1.6 | *P* ≤ 0.005 |
| *speF* | ornithine decarboxylase isozyme, inducible | -5.2 | *P* ≤ 0.005 | -4.3 | *P* ≤ 0.005 |
| *potE* | APC family, putrescine/ornithine antiporter | -4.2 | *P* ≤ 0.005 | -3.2 | *P* ≤ 0.005 |
| *thrA* | aspartokinase I , bifunctional enzyme N-terminal is aspartokinaseI and C-terminal is homoserine dehydrogenase I | -2.6 | *P* ≤ 0.005 | -0.4 | NS |
| *thrB* | homoserine kinase | -2.1 | *P* ≤ 0.005 | -0.6 | *P* ≤ 0.005 |
| *thrC* | threonine synthase | -2.4 | *P* ≤ 0.005 | -1.0 | *P* ≤ 0.005 |

Table S10. Effect of *trans*-cinnamaldehyde and eugenol on glycerol-3-phosphate metabolism genes

| Gene | Function | *Trans*-cinnamaldehyde | | Eugenol | |
| --- | --- | --- | --- | --- | --- |
|  |  | M value | P Value | M value | P value |
| *glpQ* | Glycerophosphodiester phosphodiesterase, periplasmic | -1.8 | *P* ≤ 0.005 | -2.7 | *P* ≤ 0.005 |
| *glpT* | MFS family, sn-glycerol-3-phosphate transport protein | -0.9 | *P* ≤ 0.005 | -1.9 | *P* ≤ 0.005 |
| *gldA* | Glycerol dehydrogenase | -2.7 | *P* ≤ 0.005 | -2.6 | *P* ≤ 0.005 |
| *glpK* | Glycerol kinase | -2.0 | *P* ≤ 0.005 | -1.2 | *P* ≤ 0.005 |

Table S11. Effect of *trans*-cinnamaldehyde and eugenol on genes responsible for carbon compound degradation

| Gene | Function | *Trans*-cinnamaldehyde | | Eugenol | |
| --- | --- | --- | --- | --- | --- |
|  |  | M value | P Value | M value | P value |
| *caiF* | Transcriptional regulator of *cai* and *fix* operon | -1.3 | *P* ≤ 0.005 | -1.4 | *P* ≤ 0.005 |
| *rbsC* | ABC superfamily (membrane), D-ribose high-affinity transport protein (1st module, ATP-binding subunit) | -1.3 | *P* ≤ 0.005 | -0.4 | *P* = 0.03 |
| *rbsB* | ABC superfamily, D-ribose transport protein | -2.0 | *P* ≤ 0.005 | -1.3 | *P* ≤ 0.005 |
| *rbsK* | Ribokinase | -2.1 | *P* ≤ 0.005 | -1.4 | *P* ≤ 0.005 |
| *rbsR* | Transcriptional repressor for rbs operon (GalR/LacI family) | -1.5 | *P* ≤ 0.005 | -1.5 | *P* ≤ 0.005 |

Table S12. Effect of *trans*-cinnamaldehyde and eugenol on genes responsible for tetrathionate reduction

| Gene | Function | *Trans*-cinnamaldehyde | | Eugenol | |
| --- | --- | --- | --- | --- | --- |
|  |  | M value | P Value | M value | P value |
| *ttrR* | Tetrathionate reductase complex: response regulator | -2.0 | *P* ≤ 0.005 | -1.4 | *P* ≤ 0.005 |
| *ttrS* | Tetrathionate reductase complex: sensory transduction histidine kinase | -2.2 | *P* ≤ 0.005 | -2.3 | *P* ≤ 0.005 |
| *ttrB* | Tetrathionate reductase complex, subunit B | -1.6 | *P* ≤ 0.005 | -0.8 | *P* ≤ 0.005 |
| *ttrC* | Tetrathionate reductase complex, subunit C | -0.8 | *P* ≤ 0.005 | -0.2 | NS |
| *ttrA* | Tetrathionate reductase complex, subunit A | -2.1 | *P* ≤ 0.005 | -0.7 | *P* ≤ 0.005 |

Table S13. Effect of *trans*-cinnamaldehyde and eugenol on genes responsible for propanediol reduction

| Gene | Function | *Trans*-cinnamaldehyde | | Eugenol | |
| --- | --- | --- | --- | --- | --- |
|  |  | M value | P Value | M value | P value |
| *pocR* | Propanediol utilization: transcriptional regulation, AraC family | -2.5 | *P* ≤ 0.005 | -2.2 | *P* ≤ 0.005 |
| *pduF* | Propanediol utilization: propanediol diffusion facilitator | -2.4 | *P* ≤ 0.005 | -1.4 | *P* ≤ 0.005 |
| *pduA* | Propanediol utilization: polyhedral bodies | -2.5 | *P* ≤ 0.005 | -1.9 | *P* ≤ 0.005 |
| *pudB* | Propanediol utilization: polyhedral bodies | -4.0 | *P* ≤ 0.005 | -3.5 | *P* ≤ 0.005 |
| *pduC* | Propanediol utilization: dehydratase, large subunit | -4.2 | *P* ≤ 0.005 | -3.8 | *P* ≤ 0.005 |
| *pduD* | Propanediol utilization: dehydratase, medium subunit | -4.2 | *P* ≤ 0.005 | -4.6 | *P* ≤ 0.005 |
| *pduE* | Propanediol utilization: dehydratase, small subunit | -2.1 | *P* ≤ 0.005 | -1.6 | *P* ≤ 0.005 |
| *pduG* | Propanediol utilization: diol dehydratase reactivation | -3.2 | *P* ≤ 0.005 | -3.0 | *P* ≤ 0.005 |
| *pduH* | Propanediol utilization: diol dehydratase reactivation | -1.0 | *P* ≤ 0.005 | -0.5 | *P* = 0.03 |
| *pduJ* | Propanediol utilization: polyhedral bodies | -0.8 | *P* = 0.007 | -0.8 | *P* ≤ 0.005 |
| *pduK* | Propanediol utilization: polyhedral bodies | -2.5 | *P* ≤ 0.005 | -2.6 | *P* ≤ 0.005 |
| *pduL* | Propanediol utilization | -2.5 | *P* ≤ 0.005 | -3.3 | *P* ≤ 0.005 |
| *pduM* | Propanediol utilization | -2.4 | *P* ≤ 0.005 | -2.3 | *P* ≤ 0.005 |
| *pduN* | Propanediol utilization: polyhedral bodies | -1.6 | *P* ≤ 0.005 | -1.2 | *P* ≤ 0.005 |
| *pduO* | Propanediol utilization: B12 related | -1.9 | *P* ≤ 0.005 | -1.8 | *P* ≤ 0.005 |
| *pduP* | Propanediol utilization: CoA-dependent propionaldehyde dehydrogenase | -2.6 | *P* ≤ 0.005 | -3.1 | *P* ≤ 0.005 |
| *pduQ* | Propanediol utilization: propanol dehydrogenase | -1.8 | *P* ≤ 0.005 | -2.6 | *P* ≤ 0.005 |
| *pduS* | Propanediol utilization: polyhedral bodies | -1.7 | *P* ≤ 0.005 | -2.0 | *P* ≤ 0.005 |
| *pduT* | Propanediol utilization: polyhedral bodies | -2.0 | *P* ≤ 0.005 | -2.6 | *P* ≤ 0.005 |
| *pduU* | Propanediol utilization: polyhedral bodies | -1.6 | *P* ≤ 0.005 | -2.5 | *P* ≤ 0.005 |
| *pduV* | Propanediol utilization | -1.4 | *P* ≤ 0.005 | -1.7 | *P* ≤ 0.005 |
| *pduW* | Propanediol utilization: propionate kinase | -1.9 | *P* ≤ 0.005 | -3.1 | *P* ≤ 0.005 |
| *pduX* | Propaendiol utilization | -0.5 | *P* ≤ 0.005 | -1.2 | *P* ≤ 0.005 |

Table S14. Effect of *trans*-cinnamaldehyde and eugenol on genes responsible for ethanolamine utilization

| Gene | Function | *Trans*-cinnamaldehyde | | Eugenol | |
| --- | --- | --- | --- | --- | --- |
|  |  | M value | P Value | M value | P value |
| *eutC* | Ethanolamine ammonia-lyase, light chain | -1.5 | *P* ≤ 0.005 | -0.5 | *P* = 0.02 |
| *eutB* | Ethanolamine ammonia-lyase, heavy chain | -2.1 | *P* ≤ 0.005 | -0.8 | *P* ≤ 0.005 |
| *eutH* | Putative transport protein, ethanolamine utilization | -2.1 | *P* ≤ 0.005 | -1.1 | *P* ≤ 0.005 |
| *eutG* | Paral Putative transport protein in ethanolamine utilization | -1.0 | *P* ≤ 0.005 | -0.3 | NS |
| *eutJ* | Paral Putative heatshock protein (Hsp70) | -2.1 | *P* ≤ 0.005 | -1.0 | *P* = 0.007 |
| *eutE* | Putative aldehyde oxidoreductase in ethanolamine utilization | -2.6 | *P* ≤ 0.005 | -1.6 | *P* ≤ 0.005 |
| *eutN* | Putative detox protein in ethanolamine utilization | -2.3 | *P* ≤ 0.005 | -1.4 | *P* ≤ 0.005 |
| *eutD* | Putative phosphotransacetylase in ethanolamine utilization | -1.1 | *P* ≤ 0.005 | -0.8 | NS |
| *eutT* | Putative cobalamin adenosyltransferase, ethanolamine utilization | -2.8 | *P* ≤ 0.005 | -1.6 | *P* ≤ 0.005 |
| *eutQ* | Putative ethanolamine utilization protein | -3.1 | *P* ≤ 0.005 | -1.9 | *P* ≤ 0.005 |
| *eutP* | Putative ethanolamine utilization protein | -2.1 | *P* ≤ 0.005 | -1.1 | *P* ≤ 0.005 |
| *eutS* | Putative carboxysome structural protein, ethanol utilization | -1.5 | *P* ≤ 0.005 | -1.6 | *P* ≤ 0.005 |

Table S15. Effect of *trans*-cinnamaldehyde and eugenol on genes responsible for dimethyl sulphide reduction

| Gene | Function | *Trans*-cinnamaldehyde | | Eugenol | |
| --- | --- | --- | --- | --- | --- |
|  |  | M value | P Value | M value | P value |
| *dmsB* | Anaerobic dimethyl sulfoxide reductase subunit B | -4.5 | *P* ≤ 0.005 | -3.2 | *P* ≤ 0.005 |
| *dmsA* | Putative anaerobic dimethyl sulfoxide reductase, subunit A pseudogene | -5.3 | *P* ≤ 0.005 | -3.9 | *P* ≤ 0.005 |
| *dmsC* | Putative dimethyl sulfoxide reductase subunit C | -3.0 | *P* ≤ 0.005 | -1.5 | *P* ≤ 0.005 |
| *STM1498* | Putative dimethyl sulfoxide reductase | -2.9 | *P* ≤ 0.005 | -2.1 | *P* ≤ 0.005 |
| *STM1499* | Putative dimethyl sulfoxide reductase, chain A1 | -2.9 | *P* ≤ 0.005 | -2.2 | *P* ≤ 0.005 |

Table S16. Effect of *trans*-cinnamaldehyde and eugenol on genes responsible for molybdopterin synthesis

| Gene | Function | *Trans*-cinnamaldehyde | | Eugenol | |
| --- | --- | --- | --- | --- | --- |
|  |  | M value | P Value | M value | P value |
| *moaA* | Molybdopterin biosynthesis, protein A | -0.5 | *P* ≤ 0.005 | -0.8 | *P* ≤ 0.005 |
| *moaB* | Molybdopterin biosynthesis, protein B | -1.0 | *P* ≤ 0.005 | -2.0 | *P* ≤ 0.005 |
| *moaC* | Molybdopterin biosynthesis, protein C | -0.9 | *P* ≤ 0.005 | -1.1 | *P* ≤ 0.005 |
| *moaE* | Molybdopterin converting factor, subunit 2 | -0.7 | *P* ≤ 0.005 | -0.3 | NS |
| *fdnG* | Putative molybdopterin oxidoreductases | -1.8 | *P* ≤ 0.005 | -0.7 | *P* ≤ 0.005 |

Table S17. Effect of *trans*-cinnamaldehyde and eugenol on genes responsible for vitamin B12 synthesis

| Gene | Function | *Trans*-cinnamaldehyde | | Eugenol | |
| --- | --- | --- | --- | --- | --- |
|  |  | M value | P Value | M value | P value |
| *cbiP* | Synthesis of vitamin B12 adenosyl cobalamide precursor | -1.8 | *P* ≤ 0.005 | -2.0 | *P* ≤ 0.005 |
| *cbiO* | Synthesis of vitamin B12 adenosyl cobalamide precursor | -1.0 | *P* ≤ 0.005 | -1.3 | *P* ≤ 0.005 |
| *cboQ* | Synthesis of vitamin B12 adenosyl cobalamide precursor | -2.2 | *P* ≤ 0.005 | -2.6 | *P* ≤ 0.005 |
| *cbiN* | Synthesis of vitamin B12 adenosyl cobalamide precursor | -1.7 | *P* ≤ 0.005 | -2.1 | *P* ≤ 0.005 |
| *cbiM* | Synthesis of vitamin B12 adenosyl cobalamide precursor | -2.8 | *P* ≤ 0.005 | -2.6 | *P* ≤ 0.005 |
| *cbiL* | Synthesis of vitamin B12 adenosyl cobalamide precursor | -2.7 | *P* ≤ 0.005 | -2.6 | *P* ≤ 0.005 |
| *cbiK* | Synthesis of vitamin B12 adenosyl cobalamide precursor | -1.4 | *P* ≤ 0.005 | -1.4 | *P* ≤ 0.005 |
| *cbiJ* | Synthesis of vitamin B12 adenosyl cobalamide precursor | -1.6 | *P* ≤ 0.005 | -2.2 | *P* ≤ 0.005 |
| *cbiH* | Synthesis of vitamin B12 adenosyl cobalamide precursor | -2.0 | *P* ≤ 0.005 | -2.4 | *P* ≤ 0.005 |
| *cbiG* | Synthesis of vitamin B12 adenosyl cobalamide precursor | -2.3 | *P* ≤ 0.005 | -2.5 | *P* ≤ 0.005 |
| *cbiF* | Synthesis of vitamin B12 adenosyl cobalamide precursor | -2.3 | *P* ≤ 0.005 | -2.4 | *P* ≤ 0.005 |
| *cbiT* | Synthesis of vitamin B12 adenosyl cobalamide precursor | -1.0 | *P* ≤ 0.005 | -1.4 | *P* ≤ 0.005 |
| *cbiE* | Synthesis of vitamin B12 adenosyl cobalamide precursor | -1.0 | *P* ≤ 0.005 | -1.8 | *P* ≤ 0.005 |
| *cbiD* | Synthesis of vitamin B12 adenosyl cobalamide precursor | -1.2 | *P* ≤ 0.005 | -2.0 | *P* ≤ 0.005 |
| *cbiC* | Synthesis of vitamin B12 adenosyl cobalamide precursor | -1.7 | *P* ≤ 0.005 | -1.8 | *P* ≤ 0.005 |
| *cibB* | Synthesis of vitamin B12 adenosyl cobalamide precursor | -0.9 | *P* ≤ 0.005 | -1.8 | *P* ≤ 0.005 |
| *cbiA* | Synthesis of vitamin B12 adenosyl cobalamide precursor | -2.2 | *P* ≤ 0.005 | -1.8 | *P* ≤ 0.005 |

Table S18. Effect of *trans*-cinnamaldehyde and eugenol on genes responsible for hydrogen sulphide production

| Gene | Function | *Trans*-cinnamaldehyde | | Eugenol | |
| --- | --- | --- | --- | --- | --- |
|  |  | M value | P Value | M value | P value |
| *phsC* | Hydrogen sulfide production: membrane anchoring protein | -2.6 | *P* ≤ 0.005 | -2.7 | *P* ≤ 0.005 |
| *phsB* | Hydrogen sulfide production: iron- sulfur subunit; electron transfer | -3.0 | *P* ≤ 0.005 | -2.6 | *P* ≤ 0.005 |
| *phsA* | Hydrogen sulfide production: membrane anchoring protein | -2.8 | *P* ≤ 0.005 | -3.0 | *P* ≤ 0.005 |

Table S19. List of up-regulated genes in response to *trans*-cinnamaldehyde and eugenol.

| Gene | Function | *Trans*-cinnamaldehyde | | Eugenol | |
| --- | --- | --- | --- | --- | --- |
|  |  | M value | P Value | M value | P value |
| *clpB* | ATP-dependent protease, Hsp 100, part of novel  multi-chaperone system with DnaK, DnaJ, and GrpE | 1.0 | *P* ≤ 0.005 | 1.6 | *P* ≤ 0.005 |
| *clpP* | Proteolytic subunit of clpA-clpP ATP-dependent serine  protease, heat shock protein F21.5 | 0.5 | *P* ≤ 0.005 | 1.3 | *P* ≤ 0.005 |
| *clpX* | Specificity component of clpA-clpP ATP-dependent  serine protease, chaperone | 0.7 | *P* ≤ 0.005 | 0.6 | NS |
| *dnaK* | Chaperone Hsp70 in DNA biosynthesis/cell division | 2.9 | *P* ≤ 0.005 | 3.3 | *P* ≤ 0.005 |
| *dnaJ* | Heat shock protein, DnaJ and GrpE stimulates ATPase  activity of DnaK | 2.7 | *P* ≤ 0.005 | 3.3 | *P* ≤ 0.005 |
| *rpoS* | Sigma S (sigma 38) factor of RNA polymerase, major  Sigma factor during stationary phase | 0.5 | P = 0.01 | 0.5 | NS |
| *ibpB* | Small heat shock protein | 5.8 | *P* ≤ 0.005 | 3.5 | *P* ≤ 0.005 |
| *ibpA* | Small heat shock protein | 4.1 | *P* ≤ 0.005 | 4.3 | *P* ≤ 0.005 |
| *mopB* | Chaperone Hsp10, affects cell division | 1.7 | *P* ≤ 0.005 | 2.3 | *P* ≤ 0.005 |
| *mopA* | Chaperone Hsp60 with peptide-dependent  ATPase activity, affects cell division | 2.8 | *P* ≤ 0.005 | 3.3 | *P* ≤ 0.005 |
| *sodC* | Gifsy-2 prophage: superoxide dismutase precursor (Cu-Zn) | 1.0 | *P* ≤ 0.005 | 0.6 | *P* ≤ 0.005 |
| *sodA* | Superoxide dismutase, Mn | 1.8 | *P* ≤ 0.005 | 3.2 | *P* ≤ 0.005 |
| *yacK* | Putative multicopper oxidase | 2.9 | *P* ≤ 0.005 | 2.9 | *P* ≤ 0.005 |
| *yafH* | Putative acyl CoA dehydrogenase | 4.1 | *P* ≤ 0.005 | 4.9 | *P* ≤ 0.005 |
| *acrA* | Acridine Efflux Pump | 1.2 | *P* ≤ 0.005 | 2.1 | *P* ≤ 0.005 |
| *acrB* | Acridine Efflux Pump | 1.0 | *P* ≤ 0.005 | 2.0 | *P* ≤ 0.005 |
| *acrR* | acrAB operon repressor | 1.1 | *P* ≤ 0.005 | 1.2 | *P* ≤ 0.005 |
| *htpG* | Chaperone Hsp9 | 3.2 | *P* ≤ 0.005 | 4.0 | *P* ≤ 0.005 |
| *citC* | Citrate lyase synthetase | 0.5 | NS | 2.3 | *P* ≤ 0.005 |
| *citD* | Citrate lyase acyl carrier protein | 0.9 | *P* ≤ 0.005 | 2.4 | *P* ≤ 0.005 |
| *citE* | Citrate lyase beta chain | 0.7 | NS | 2.9 | *P* ≤ 0.005 |
| *citF* | Bifunctional citrate lyase alpha chain | 0.6 | NS | 2.7 | *P* ≤ 0.005 |
| *ybiJ* | Putative periplasmic protein | 2.9 | *P* ≤ 0.005 | 4.1 | *P* ≤ 0.005 |
| *ycfJ* | Putative outermembrane lipoprotein | 2.7 | *P* ≤ 0.005 | 3.0 | *P* ≤ 0.005 |
| *ycfR* | Putative outermembrane lipoprotein | 4.7 | *P* ≤ 0.005 | 3.7 | *P* ≤ 0.005 |
| *marB* | Mutliple antibiotic resistance protein | 3.4 | *P* ≤ 0.005 | 1.0 | *P* ≤ 0.005 |
| *marA* | Transcriptional Activator | 5.5 | *P* ≤ 0.005 | 4.5 | *P* ≤ 0.005 |
| *marR* | Transcriptional repressor of *marRAB* operon | 3.7 | *P* ≤ 0.005 | 5.9 | *P* ≤ 0.005 |
| *marC* | Putative marC transporter | 1.3 | *P* ≤ 0.005 | 1.5 | *P* ≤ 0.005 |
| *fadD* | Acyl CoA synthetase | 2.9 | *P* ≤ 0.005 | 2.8 | *P* ≤ 0.005 |
| *fadL* | Transport of long-chain fatty acid | 3.9 | *P* ≤ 0.005 | 1.2 | *P* ≤ 0.005 |
| *yhcN* | Putative outermembrane protein | 3.6 | *P* ≤ 0.005 | 3.1 | *P* ≤ 0.005 |
| *yhdV* | Putative outermembrane lipoprotein | 0.7 | *P* ≤ 0.005 | 2.4 | *P* ≤ 0.005 |
| *fadA* | 3-keto acyl CoA thiolase | 2.9 | *P* ≤ 0.005 | 2.9 | *P* ≤ 0.005 |
| *fadB* | 3 hydroxyacyl CoA dehydrogenase | 2.7 | *P* ≤ 0.005 | 3.4 | *P* ≤ 0.005 |
